# To Know: Its Source, Its Mode, and Its Power

**Published:** 1864-03

**Authors:** 


To Know: Its Source, its Mode, and its Power. An Introductory Address, Delivered at the St. Louis Medical College, November 2d, 1863. By Frank. W. White, A.M., M.D., Professor of Materia Medica and Therapeutics.

This is a well-written address, earnest and elevated in its tone, and admirably calculated to excite activity and a proper emulation in those to whom it was addressed.

